# Telemedicine for Prescribing Nirmatrelvir/Ritonavir: Safety, Logistics, and Challenges

**DOI:** 10.1093/ofid/ofae283

**Published:** 2024-05-14

**Authors:** Tyler Liebenstein, Miguel Mailig, Christopher J Crnich, Prakash Balasubramanian

**Affiliations:** Department of Pharmacy, William S. Middleton Memorial Veterans Hospital, Madison, Wisconsin, USA; Department of Pharmacy, William S. Middleton Memorial Veterans Hospital, Madison, Wisconsin, USA; Department of Medicine, William S. Middleton Memorial Veterans Hospital, Madison, Wisconsin, USA; Department of Medicine, University of Wisconsin Health, Madison, Wisconsin, USA; Department of Medicine, William S. Middleton Memorial Veterans Hospital, Madison, Wisconsin, USA

**Keywords:** COVID-19, drug interactions, interdisciplinary team, Paxlovid, telemedicine

## Abstract

Nirmatrelvir/ritonavir can be a useful COVID-19 treatment but is challenging to prescribe safely because of drug-drug interactions. This study describes our experience prescribing nirmatrelvir/ritonavir within a small interdisciplinary team with a specific focus on management of drug-drug interactions. Ascertaining and communicating modifications of concomitant medications is a key safety element.

## Teaser

Nirmatrelvir/ritonavir is challenging to prescribe safely because of drug-drug interactions. In this study, an interdisciplinary team was able to prescribe nirmatrelvir/ritonavir safely and feasibly via telemedicine. Providers should be diligent about ascertaining and communicating modifications of concomitant medications to patients.

Nirmatrelvir/ritonavir (NRM/r) is approved by the US Food and Drug Administration for the treatment of COVID-19 infection [1, 2]. Real-world studies support use with decreases in hospitalizations and mortality in those treated with NRM/r [3–6]. One of the major drawbacks of NRM/r is the potential for drug-drug interactions (DDIs) with concurrent medications. NRM/r is expected to interact with 30 of the top 100 most prescribed medications among those at increased risk for severe COVID [2, 7, 8] Although there are simulations of the extent of DDIs that providers might encounter, details regarding magnitude of DDIs encountered in clinical practice are sparse [9, 10].

Though unfamiliar to most providers, prescribing NRM/r via telemedicine quickly became the preferred route given the need to minimize in-person contact. Prescribing NRM/r via telemedicine poses unique challenges given the extent of DDIs, modification of concurrent medications, renal dosing, and other requirements. Provider discomfort with prescribing NRM/r because of DDIs remains a significant issue [11].

In this analysis, we describe our institution's experience with prescribing NRM/r via telemedicine, focusing on the safety of DDI management. Our facility is a Veterans Affairs medical center that consists of a hospital providing tertiary care and 6 community-based clinics serving 95 000 veterans. All patients with symptoms concerning for COVID were directed to a team of COVID care nurses who facilitated testing, provided advice on symptomatic treatment, isolation, and masking. Patients who tested positive for COVID-19, were high-risk, and within the window of treatment were screened by nurses for severity of illness and subsequently referred to a dedicated COVID therapeutics provider. The team was led by an infectious diseases (ID) physician and an ID pharmacist. Initially, the providers consisted of hospitalists but were ultimately transitioned to a group of approximately 15 primary care nurse practitioners and physician assistants. ID providers were available to provide clarifications when needed. This team was available primarily during business hours, but after hours and weekend prescribing was possible via on-call ID provider.

Before contacting the patient, the provider consulted an ID pharmacist for DDI evaluation. Resources used by the pharmacist included a DDI checker maintained by the University of Liverpool, drug package inserts, and recommendations from the US Department of Health and Human Services. Because the centralized pool of providers and pharmacists was small, this made them well-versed in navigating the nuances of NRM/r. Specialty services were consulted when modifications to condition-specific prescribed medications were deemed necessary but that could substantially impact efficacy or safety. The medical provider was responsible for confirming the active medication list and communicating changes that needed to be made to other medications to the patient. All patients prescribed NRM/r received follow-up calls by a nurse to evaluate illness trajectory and record any self-reported adverse drug reactions (ADRs).

## METHODS

We performed a retrospective review of patients prescribed NRM/r via telemedicine encounter at our institution between March 2022 and January 2023. Records reviewed included indications for receiving NRM/r, medications that could potentially interact and that required dose adjustment, time spent by provider and pharmacists, as well as ADRs. Hospitalization within 14 days following NRM/r receipt was recorded. Patients unable to receive NRM/r, predominantly because of DDIs, were excluded. This study was approved by the William S. Middleton Memorial Veterans Hospital Research and Development Committee and the University of Wisconsin Health Sciences institutional review board.

## RESULTS

**Patient Demographics and Risk Factors:** During the study period, 457 patients were prescribed NRM/r via telemedicine. Average patient age was 67.4 ± 13.4 years (median, 71.6 years) and 409 patients (89.5%) were male. Table 1 contains risk factors warranting treatment with NRM/r. A total of 327 patients (71.6%) had more than 1 risk factor for progression to severe disease and 183 patients (40.0%) had 3 or more risk factors.

**Table 1.** Baseline Demographics and Risk Factors

High-risk Condition	Number of Patients (%)
Age 65 y or greater	304 (66.5%)
Immunocompromised	43 (9.4%)
Diabetes	137 (30.0%)
Chronic heart disease	141 (30.9%)
Chronic lung disease	105 (23.0%)
Chronic kidney disease	51 (11.2%)
Obesity (body mass index >30 kg/m^2^)	266 (58.2%)
1 high-risk condition	123 (26.9%)
2 high-risk conditions	144 (31.5%)
3 high-risk conditions	113 (24.7%)
4 or more high-risk conditions	70 (15.3%)
0-5 active medications	101 (22.1%)
6-10 active medications	175 (38.3%)
11-15 active medications	99 (21.7%)
16 or greater active medications	82 (17.9%)

**Medication and DDI analysis:** Mean number of active medications on the patient's chart at the time of being prescribed NRM/r was 10.3 ± 6.3 medications (median, 9). A total of 181 patients (39.6%) had 11 or more active medications at the time NRM/r prescription (Table 1).

A pharmacist performed prospective DDI review for 355 patients (77.7%) and spent an average of 14.1 minutes providing and documenting recommendations (range, 4-45; median, 15). Thirty-six patients (7.9%) were reviewed by an ID physician and 66 patients (14.4%) were reviewed by the prescribing provider.

The average patient had 2 potential DDIs and 394 patients (86.2%) had at least 1 potential DDI between NRM/r and concomitant medications (Table 2). Potential DDIs with 2 or more drugs occurred in 274 patients (60%). On average, 1.5 DDIs required dose modification per patient and 360 patients (78.8%) required at least 1 drug modification. Two or more drugs required modification in 204 patients (44.6%). Statins, calcium channel blockers, alpha blockers, phosphodiesterase 5 inhibitors, anticoagulants, opioids, and salmeterol were the most encountered DDIs.

**Table 2.** Drug-drug Interactions With Nirmatrelvir/Ritonavir

Number of Medications Interacting With Nirmatrelvir/ritonavir	Number of Patients (%)	Number of Medications Requiring Modification	Number of Patients (%)
0	63 (13.8%)	0	97 (21.2%)
1	120 (26.3%)	1	156 (34.1%)
2	118 (25.8%)	2	128 (28.0%)
3	86 (18.8%)	3	52 (11.4%)
4	37 (8.1%)	4	15 (3.3%)
>/=5	33 (7.2%)	≥5	9 (2.0%)

Common drugs and drug classes interacting with nirmatrelvir/ritonavir	Number of patients (%)	Number of patients needing dose modification
Statin	235 (51.4%)	234 (99.6%)
Alpha blocker	110 (24.1%)	77 (70%)
Calcium channel blocker	93 (20.4%)	86 (92.5%)
Phosphodiesterase-5 inhibitor	67 (14.7%)	65 (97.0%)
Anticoagulant	46 (10.1%)	41 (89.1%)
Opioid	30 (6.6%)	13 (43.3%)
Trazodone	32 (7.0%)	27 (84.4%)
Salmeterol	25 (5.5%)	25 (100%)

Specialty services provided DDI management advice for 47 patients (10.3%). Most commonly, this was the anticoagulation clinic (40 patients), but also included cardiology, hematology, and rheumatology.

Estimated glomerular filtration rate was available for 456 of 457 patients (99.8%), obtained an average of 4.5 months (range, 0-57; median, 3) before telemedicine, encounter which was deemed sufficient for prescribing purposes. No patient was directed for an in-person visit primarily to obtain updated estimated glomerular filtration rate.

On average, providers spent 22.6 minutes in the telemedicine encounter, including medical record review, patient interaction, and documentation.

**Adverse Reactions and Hospitalizations:** Potential treatment related ADRs were identified in 162 patients (35.4%); altered taste (n = 79, 17.3%) and diarrhea (n = 68, 14.4%) were the most commonly reported. Twelve patients (2.6%) prescribed NRM/r discontinued the medication because of ADR.

Fifteen patients (3.3%) required hospitalization for any reason within 14 days of NRM/r prescription. Two patients (0.4%) were admitted to the hospital for symptoms deemed potentially related to NRM/r ADR. Patient 1 was admitted for hypotension in the setting of confusion regarding concomitant medications to hold. The patient was thought to have continued amlodipine and tamsulosin without making modifications as suggested. Patient 2 was admitted with a possible sequela of COVID infection rather than NRM/r ADR, though this could not be ruled out conclusively. Two patients died during the study period; neither death was deemed related to NRM/r nor COVID.

## CONCLUSIONS

Prescribing COVID therapeutics via telemedicine has been used increasingly as a strategy to promote treatment access. However, this can add a layer of complexity to the already challenging task of prescribing NRM/r. Our study highlights the challenges, particularly regarding DDI evaluation and management, when prescribing NRM/r via telemedicine.

Before interacting with patients, providers should preview the electronic health record for concomitant medications with potential for DDIs and determine those that will need to be held or have the dose adjusted. In our study, the average patient had 2 medications with potential NRM/r DDIs and 1.5 medications that ultimately needed to be modified. At our facility, a pharmacist prospectively reviewed most patients (77.7%) for DDIs, helping the provider be prepared for the telemedicine encounter. In our facility, the pharmacist had full electronic health record access, which may not reflect all sites of NRM/r prescribing.

Following DDI review, telemedicine evaluation includes ensuring the patient is within the recommended treatment window and the patient's symptoms are mild enough to safely prescribe NRM/r via telemedicine rather than an in-person visit. When prescribing NRM/r via telemedicine, providers should pay special attention to communicating medication changes that need to be made. In our study, 78.8% of patients needed to make at least 1 medication adjustment when receiving NRM/r, with 44.6% needing 2 or more modifications. Providers should ensure patients understand these changes or speak with a caregiver that can assist. Pharmacies may not always have access to all the required health information to communicate the necessary changes.

Providers spent an average of 22.6 minutes in prescribing NRM/r via telemedicine in our study, although the time spent can vary. This is in addition the time spent by pharmacists (14.1 minutes) on DDI evaluation. Clinic schedulers will need to allow adequate time for providers to evaluate and treat COVID-positive patients and be able to accommodate varying lengths of a telemedicine visit.

Although the process can be complex, prescribing NRM/r via telemedicine visit appeared safe in our study. While 35.4% of patients reported an ADR, most were minor and related to altered taste or gastrointestinal upset. Only 2.6% of patients discontinued NRM/r because of ADR.

Our study does have important limitations. First, only patients who were prescribed NRM/r via telemedicine were included. Other patients with COVID-19, including those given other treatment modalities, those contraindicated for NRM/r, those not given any treatment, and those prescribed NRM/r in-person via the emergency department or urgent care were not included. Second, our study population was performed in a closed health care system, which may not apply to other sites of NRM/r prescribing.

Our study demonstrates that prescribing NRM/r via telemedicine visit can be done safely and serve as an efficient modality of providing timely care for COVID-positive patients at high risk for progression to severe disease. However, the process is complex and requires coordinated efforts from interdisciplinary team members.

## Notes

***Acknowledgments*.** The authors thank Brittney Obi and the team of COVID After Care nurses as well as Jonathan Hopman and the team of COVID treatment providers for services rendered in caring for patients with COVID and in prescribing nirmatrelvir-ritonavir.

***Patient Consent Statement*.** This study does not include factors necessitating patient consent. This study was approved by the William S. Middleton Memorial Veterans Hospital Research and Development Committee and the University of Wisconsin Health Sciences institutional review board.

***Financial support*.** There was no funding that supported this work.
